# Catalyzed Ester Synthesis Using *Candida rugosa* Lipase Entrapped by Poly(*N*-isopropylacrylamide-*co*-itaconic Acid) Hydrogel

**DOI:** 10.1155/2014/142123

**Published:** 2014-02-20

**Authors:** Nikola Milašinović, Sonja Jakovetić, Zorica Knežević-Jugović, Nedeljko Milosavljević, Marija Lučić, Jovanka Filipović, Melina Kalagasidis Krušić

**Affiliations:** ^1^Department of Criminalistic Sciences, The Academy of Criminalistic and Police Studies, Cara Dušana 196, 11080 Belgrade, Serbia; ^2^Department of Biochemical Engineering and Biotechnology, Faculty of Technology and Metallurgy, University of Belgrade, Karnegijeva 4, 11000 Belgrade, Serbia; ^3^Department of Organic Chemical Technology, Faculty of Technology and Metallurgy, University of Belgrade, Karnegijeva 4, 11000 Belgrade, Serbia

## Abstract

This study reports the synthesis of polymeric matrices based on *N*-isopropylacrylamide and itaconic acid and its application for immobilization of lipase from *Candida rugosa*. The lipase was immobilized by entrapment method. Free and immobilized lipase activities, pH and temperature optima, and storage stability were investigated. The optimum temperature for free and entrapped lipase was found to be 40 and 45°C, while the optimum pH was observed at pH 7 and 8, respectively. Both hydrolytic activity in an aqueous medium and esterolytic activity in an organic medium have been evaluated. Maximum reaction rate (*V*_max_) and Michaelis-Menten constants (*K*_*m*_) were also determined for immobilized lipase. Storage stability of lipase was increased as a result of immobilization process. Furthermore, the operational stability and reusability of the immobilized lipase in esterification reaction have been studied, and it was observed that after 10 cycles, the residual activity for entrapped lipase was as high as 50%, implying that the developed hydrogel and immobilized system could provide a promising solution for the flavor ester synthesis at the industrial scale.

## 1. Introduction

Lipase-catalyzed esterification reactions in organic solvents are of interest during the last decade. However, the systems formed by amphiphilic networks have not been widely studied as tools for enzyme reactions in hydrophobic solvents [1, 2]. In such systems, the interfacial active enzyme, such as lipase, can effectively catalyze not only hydrolytic but also synthetic reactions [3, 4].

However, the purification of the enzyme as a catalyst is expensive and complex and, due to its instability in solution, it loses its activity after few hours. Despite their advantages, biocatalysts are not usually used in industrial processes for their high cost, difficulty of the product purification, and the instability of the enzymes which limits their reuse.

Among different ways to improve their performance as catalysts, the immobilization into a suitable solid support, by combining an enzyme with a polymeric support, would allow their reuse and facilitate the separation of the products, thus generating an economically viable bioconversion process technology [5–7]. Recently, hydrogels have attracted a considerable attention due to their potential applications as matrices for enzyme immobilization. These catalytic conjugates containing lipase molecules have been reported as effective catalysts for fatty acid esterification [8–10].

Immobilized enzymes are protected by the solid matrix that limits their conformational changes diminishing variations of their properties. The immobilization of enzymes can be performed by different methods on a variety of supports. Therefore, the enzymes can be adsorbed on insoluble polymeric materials, encapsulated in gels, crosslinked with a bifunctional reagent, and covalently coupled or entrapped within an insoluble polymeric matrix as hydrogel [5, 11–14].

The three-dimensional network can create an adequate and protective microenvironment for the enzyme protection and stability, enhanced by the involved support-enzyme interaction forces, such as electrostatic and hydrophobic interactions, that greatly influence the enzyme performance as biocatalyst. In the last years, several research groups studied the immobilization of the *Candida rugosa* lipase enzyme onto hydrogels [13–21]. The hydrogels sensitive to temperature and/or pH of the surroundings are very interesting considering efficiency of immobilization and the ease of handling, which can be useful for the immobilization of the enzyme on the hydrogel, as well as for application of the immobilized enzyme as a biocatalyst.

This paper reports the synthesis of a biocatalyst using lipase from *Candida rugosa *(CRL) absorbed onto previously synthetized support, temperature- and pH-sensitive poly(*N*-isopropylacrylamide-*co*-itaconic acid) hydrogel, P(NiPAAm/IA), and its application in esterification synthesis of *n*-amyl isobutyrate. The amphiphilic character and the presence of surface groups allows interaction with proteins and physical entrapment [22, 23]. Since the reaction conditions for hydrolytic and esterification reactions are often different, a one-step-at-a-time method was employed to optimize various reaction parameters for the particular ester synthesis, such as temperature, pH, added water content and biocatalyst mass. Finally, the operational stability of lipase activity and the reusability study have been evaluated.

## 2. Experimental

### 2.1. Materials

Lipase from *Candida rugosa* (CRL) (with nominal specific lipolytic activity of 1468 IU/mg_solid_) was obtained from Sigma-Aldrich Chemie Gmbh (Germany). The monomers used in this study, *N*-isopropylacrylamide (NiPAAm) and itaconic acid (IA), were obtained from Acros Organics (Belgium). The crosslinking agent *N*,*N*′-methylenebisacrylamide (MBA) was obtained from Serva Feinbiochemica (Germany). Potassium persulphate (PPS) and potassium pyrosulphate (PPyroS), the initiator and accelerator, were obtained from Merck & Co. Inc. (Germany) and Acros Organics (Belgium), respectively. NiPAAm was recrystallized from benzene/*n*-hexane mixture (35/75) before use. Other materials were used as received, without purification. Distilled water was used for all copolymerizations and the preparation of the buffer solutions. Sodium dihydrogen phosphate dihydrate, di-sodium hydrogen phosphate dodecahydrate (Lach-Ner, s.r.o., Czech Republic), and H_3_PO_4_ (Fluka, Germany) were used to prepare the aqueous media of different pH values. Molar concentrations of all buffer solutions used were 0.2 M. Furthermore, isobutyric acid kindly purchased from Fluka Chemie GmbH (Germany) and  *n*-amyl alcohol obtained from La Chema (Czech Republic) were used for ester synthesis performed in *n*-hexane obtained from Carlo Erba Reagenti (Italy).

### 2.2. Synthesis of Hydrogel and Preparation of CRL-P(NiPAAm/IA) Biocatalyst

Free radical crosslinking copolymerization was performed at 25°C in the nitrogen atmosphere, using distilled water as a solvent. The monomers were separately dissolved in solvent. Redox couple of PPS and PPyroS, in an amount of 1.0 wt.% with respect to monomers, was added to the IA solution, prior to polymerization. The concentration of the crosslinking agent was 2.0 and 4.0 wt.%, with respect to the monomers. The reaction mixture was poured between two glass plates (21 × 6 × 0.4) cm, sealed with a PVC spacer (0.3 cm thick). After the completion of the reaction, the gels were cut into discs and flashed with water daily for a week in order to remove unreacted monomers. The discs were dried at room temperature to xerogels (0.10 ± 0.01 cm thick and 0.70 ± 0.10 cm in diameter). A model enzyme, lipase from *Candida rugosa,* was immobilized onto hydrogels upon immersing xerogels into various CRL solutions. The Lowry protein assay [24] was used to determine the loaded lipase amount as the most widely used method to estimate the amount of proteins in biological samples. The samples were left in proper CRL solutions to swell to equilibrium in order to acheive maximum lipase entrapment.

### 2.3. The Entrapment Efficiency and Activity Assays of Free and Entrapped CRL

The hydrolytic activities of free and entrapped lipase were determined according to standard olive oil emulsion procedure [25] with slight modifications. The test tubes were filled with 3.0 mL of Sigma lipase substrate (purchased from Sigma Chemical Co. (St. Louis, MO), 1.0 mL 0.05 M tris-HCl buffer solution pH 7.77, and 2.5 mL of distilled water for every single experiment. The mixtures were well stirred and left to incubate at 37°C for 20 min. In the mean time, all hydrogels were crumbled into fine powder. For the activity measurements, the proper mass of the sample was placed in the test tube, stirred again, and brought back to the thermostat for 3 hours. The blind test was done, too. The enzyme reaction was terminated by adding 3 mL of methanol/phenolphthalein mixture and the samples were titrated with 0.1 M NaOH solution to the colour change. Activities are expressed in international units (IU), defined as the amount of enzyme required to produce 1 *μ*mol of free fatty acids per minute in strictly controlled conditions (pH 7.7 and temperature of 37°C). The efficiency of immobilization was evaluated in terms of activity immobilization yield (AY) as follows:

(1) $$\begin{array}{c} \text{AY}\left(\%\right)=\frac{\text{S}{\text{A}}_{2}}{\text{S}{\text{A}}_{1}}\times 100, \end{array}$$

where SA_1_ is the specific activity of free lipase and SA_2_ is the specific activity of immobilized lipase.

Parameters such as reaction temperature, water content, pH of the reaction media, and the enzyme/support (biocatalyst) content have been optimized. The esterification was performed in screw-capped flasks in *n*-hexane. The reaction mixture containing different quantities of the biocatalyst (milled sample), various molar ratios of substrates (isobutyric acid and *n*-amyl alcohol), and different volumes of the solvent (water or buffer solutions) was diluted up to the volume of 10 mL with anhydrous *n*-hexane. The mixture was shaken at the same shaking mode for the all enzymatic reactions of 150 rpm at different temperatures in a shaking incubator. Esterification reactions were monitored by determination of the residual acid content by titration against standard 0.1 M sodium hydroxide using phenolphthalein as an indicator and methanol as a quenching agent.

## 3. Copolymer Characterization

### 3.1. Swelling Behavior

Swelling of the hydrogels prior to lipase entrapment was gravimetrically monitored in *n*-hexane and at temperature of 45°C in proper time intervals. The degree of swelling was calculated according to the following equation:

(2) $$\begin{array}{c} q=\frac{m_{t}}{m_{0}}, \end{array}$$

where *m*_0_ represents the xerogel mass and *m*_*t*_ represents the mass of the hydrogel in time *t*.

### 3.2. Fourier Transform Infrared Spectroscopy

Samples of free lipase and entrapped lipase were submitted to FT-IR analysis and the spectra were obtained using a Bomem MB 100 FT-IR Spectrophotometer. The amount of 2 wt% of the sample was mixed and ground with 100 wt% of potassium bromide and then compressed into a pellet under a pressure of 11 to 13 mm flat punch and die set, for about a minute, using Graseby Specac Model: 15.011. Spectra were obtained in the 4000–400 cm^−1^ wave number range at 25°C and at 4 cm^−1^ spectral resolution.

### 3.3. Dependence of Enzyme Activity on pH and Temperature

The effect of pH on the activity of free and entrapped lipase was investigated and determined after enzyme incubation at 45°C and in buffers solutions of different pH values ranging from 4 to 10, in a time interval of 180 minutes. The effect of temperature of free and entrapped lipase was determined after enzyme incubation at different temperatures in the range between 5 and 65°C and at pH = 7.00 for the same time period of 180 minutes.

### 3.4. The Synthesis of *n*-Amyl Isobutyrate

The esterification was performed in screw-capped flasks in *n-*hexane. The reaction mixture contained 0.6 g of the milled biocatalyst, various quantities of isobutyric acid, and *n*-amyl alcohol and 200 *μ*L of 7.00 ± 0.01 buffer solution. Each reaction mixture was diluted up to the volume of 10 mL with anhydrous *n*-hexane and put into incubating shaker (WB Memmert 22, Germany) at 150 rpm. The reaction temperature was constant (45°C). At 1 h, 2 h, 3 h and 24 h time intervals, 200 *μ*L of the reaction mixture was transferred in flasks filled with 9.80 mL of *n*-hexane and 10 mL of methanol/phenolphthalein mixture as the quenching agent. Esterification reactions were monitored by determination of the residual acid content by titration against standard 0.1 M sodium hydroxide.

### 3.5. The Kinetic Study of *n*-Amyl Isobutyrate

In order to find kinetic model of *n-*amyl isobutyrate synthesis, catalyzed with entrapped CRL, series of experiments were performed at previously determined optimal conditions. Initial reaction rates were determined for various concentrations of both substrates, where *n*-amyl alcohol was varied in the range between 0.15 and 2.35 mol/dm^3^ and isobutyric acid in the range between 0.15 and 2.40 mol/dm^3^. The experimentally obtained data were fitted with different models for bisubstrate reactions and the best fit was obtained when the ping-pong bi-bi model with alcohol inhibition was used. Equation describing this model is [26, 27]

(3) $$\begin{array}{c} v_{0}=\frac{V_{\max}\left[A_{c}\right]\left[A_{l}\right]}{{Km}_{\text{al}}\left[A_{c}\right]+{Km}_{\text{ac}}\left[A_{l}\right]\left(1+\left(\left[A_{l}\right]/K_{\text{ial}}\right)\right)+\left[A_{l}\right]\left[A_{c}\right]}, \end{array}$$

where *v*_0_ is the initial reaction rate, *V*_max⁡_ is the maximum reaction rate, *Km*_al_ and *Km*_ac_ are Michaelis constants for *n*-amyl alcohol and isobutyric acid, and *K*_ial_ is the *n*-amyl alcohol inhibition constant.

### 3.6. The Storage Stability Lipase

Dried, milled samples of biocatalysts were incubated in a laboratory freezer at 20°C, 4°C, and 25°C. Storage stability was determined over free and entrapped lipase activities in appropriate time intervals (up to 60 days for entrapped lipase), according to the previously described Sigma procedure, and results are presented as percentage of remaining activity relative to the initial biocatalysts activity, that is, relative biocatalysts activity.

### 3.7. Reusability of the Entrapped Lipase

From the economic viewpoint, the reusability of entrapped lipase is the main advantage of biocatalysts preparation. After the first esterification cycle of entrapped lipase, biocatalyst was filtered, thoroughly washed with 50 mL of *n*-hexane, and dried at 25°C for 24 hours before being subjected to repeated use and another cycle. The process of washing and reuse of entrapped lipase was performed in 20 cycles.

## 4. Results and Discussion

The effect of temperature, pH, and concentration of lipase in solution on the mass and the activity of the entrapped lipase was monitored (see Supplementary Tables 1–3 in the Supplementary Material available online at http://dx.doi.org/10.1155/2014/142123), respectively. Based on previous research [28], the adopted optimal time for conducting the reaction was 3 h. Previously synthesized xerogels with no enzyme being entrapped were left to swell to equilibrium in a lipase solution of pH 7.00 ± 0.01 and concentration of 1.0 mg_enz_/mL of at different temperatures: 5, 25, and 37°C to ensure the maximum possible entrapment of CRL onto the hydrogel matrices.

The achieved values (Supplementary Table  1) pointed that the largest percentage of specific activities and activity yields was revealed by the sample with the highest content of itaconic acid (15.0 wt.%) and less crosslinking agent content (2.0 wt%). Also, lowering the temperature at which the lipase entrapment was performed affected the increase of the investigated values, obtaining the highest values at 5°C (probably as a result of increased swelling of hydrogels at this temperature and ability to facilitate the lipase entrapment onto hydrogel). Based on previously tested mechanical properties, the hydrogels with the highest content of itaconic acid showed slightly weaker mechanical properties. Concerning these results of lipase activities, sample 90/10/2/0 was selected as potentially the best to be applied as biocatalysts in the reactions of ester synthesis.

Bearing the obtained results and the activity yields, the adopted optimal temperature for further work was 5°C. After that, the hydrogel swelling was investigated in various pH buffers (pH 6.00 ± 0.01, 8.00 ± 0.01, and 9.00 ± 0.01). The aim was to determine the optimal pH of the solution, where the activity yields show the highest values, which is important for potential use as biocatalysts in esterification reactions (Supplementary Table 2).

These results confirmed that the pH of the lipase solution in which the lipase entrapment was performed affected the yield of its activities. With increasing pH value of solution to pH 7.00, lipase activity yields increased and then, by further increase of pH solution value, decreased. Following the adoption of the optimal temperature, the optimal pH of 7.00 ± 0.01 was adopted, too. Furthermore, a set of experiments in which the lipase concentration varied between 1.0, 5.0, 10.0, and 20.0 mg_enz_/mL solution was performed (Supplementary Table 3).

With increasing concentration of lipase in solution to 10.0 mg/mL, the activity yields values increased and then slightly decreased. However, at the lipase concentration of 10 mg/mL and higher, protein deposition leads to the reduction of the lipase content that can be absorbed by the hydrogels.

In addition, besides the lipase concentration, the ratio of monomer and crosslinking agent content must be optimized, since it determines the porosity of the polymer matrices and thus affects the lipase entrapment. In this paper, it was shown that with increasing the concentration of the crosslinking agent from 2.0 to 4.0 wt.%, the activity yield reduces. For example, the activity yield of the sample 90/10/2/0 swollen under optimized conditions was 30.0%, using the standard olive oil emulsion procedure [17]. The increase in the crosslinking agent concentration reduces the number of amide groups that remain free to bind lipase after the polymerization process.

### 4.1. FT-IR Analysis

**Figure 1:**
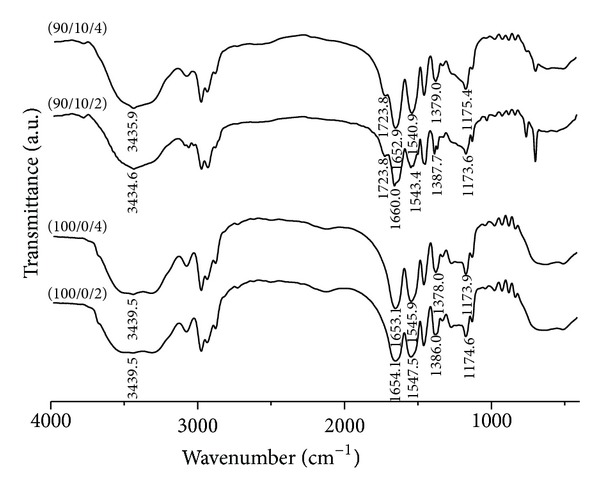
FT-IR spectra of the hydrogels prior to CRL entrapment.

The FT-IR spectra of homo- and copolymer hydrogels of different composition and degree of crosslinking were recorded and were shown in Figure 1.

FT-IR spectra of hydrogels are similar. Each spectrum shows a broad band in the wavenumber range between 3700 and 3100 cm^−1^ corresponding to OH stretching vibrations of carboxylic groups of itaconic acid and NH stretching vibration NiPAAm. Peak at 1720 cm^−1^ denotes the typical vibrations of carbonyl groups of itaconic acid [29]. The characteristic amide I and amide II bands of NiPAAm occur at 1650 cm^−1^ and 1540 cm^−1^, respectively. Two typical CH vibration bands, of almost the same intensity, at 1386 and 1379 cm^−1^ belong to the divided bands of symmetric CH(CH_3_)_2_ group. A band at 1174 cm^−1^ represents C–C stretching of CH(CH_3_)_2_ group [30].

### 4.2. Entrapped Lipase Storage Stability

**Figure 2:**
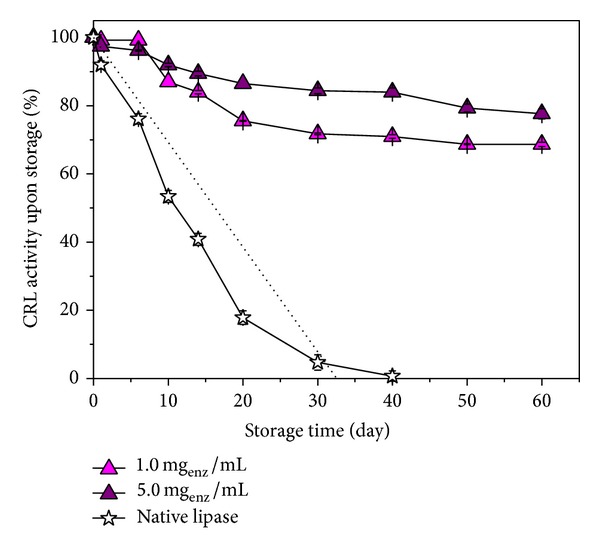
Relative activity (%) of free and entrapped lipase (for 90/10/2/0 hydrogel) upon storage at 4°C during 60 days. Reactions were performed on a standard olive oil emulsion Sigma procedure at 37°C and in pH 7.0 for 3 h.

The stability of entrapped lipase at 4°C was investigated and the results are shown in Figure 2. It was found that lipase entrapment increased the stability of lipase in comparison to native lipase. The temperature of 4°C proved suitable for entrapped lipase storage, when entrapped lipase retained about 68.7% (when entrapped in 1.0 mg_enz_/mL) and 77.0% (when entrapped in 5.0 mg_enz_/mL) of its activity. On the other hand, the free lipase retained about 41% of its catalytic activity after 14 days and only 5% after 30 days of storage at 4°C.

The lower storage temperature (−20°C) allows long-term preservation of entrapped lipase activity, with very modest possibility of degradation. The increase in storage temperature to 25°C leads to a decrease in a lipase activity, usually as a result of contamination by microorganisms. The trend line (Figure 2) provides that storage half-life of free lipase is about 16 days.

### 4.3. Optimization of Parameters of Esterification Reaction Catalyzed by Lipase from *Candida rugosa*

The time required for CRL entrapment onto hydrogels has been optimized. It was found that the activity of the entrapped lipase after 24 h was almost constant. The ability of the entrapped lipase onto P(NiPAAm/IA) hydrogel that catalyzes the synthesis of ester was examined in the reaction of synthesis of *n*-amyl isobutyrate in *n*-hexane at 45°C. The effect of various parameters such as pH, temperature, water content and the content of biocatalysts has been varied, while acid/alcohol molar ratio for the esterification reaction was kept constant (0.25 M). The reaction time of the ester synthesis was 24 h.

### 4.4. Effect of Temperature and pH on the Lipase Catalytic Activity of and Ester Synthesis

**Figure 3:**
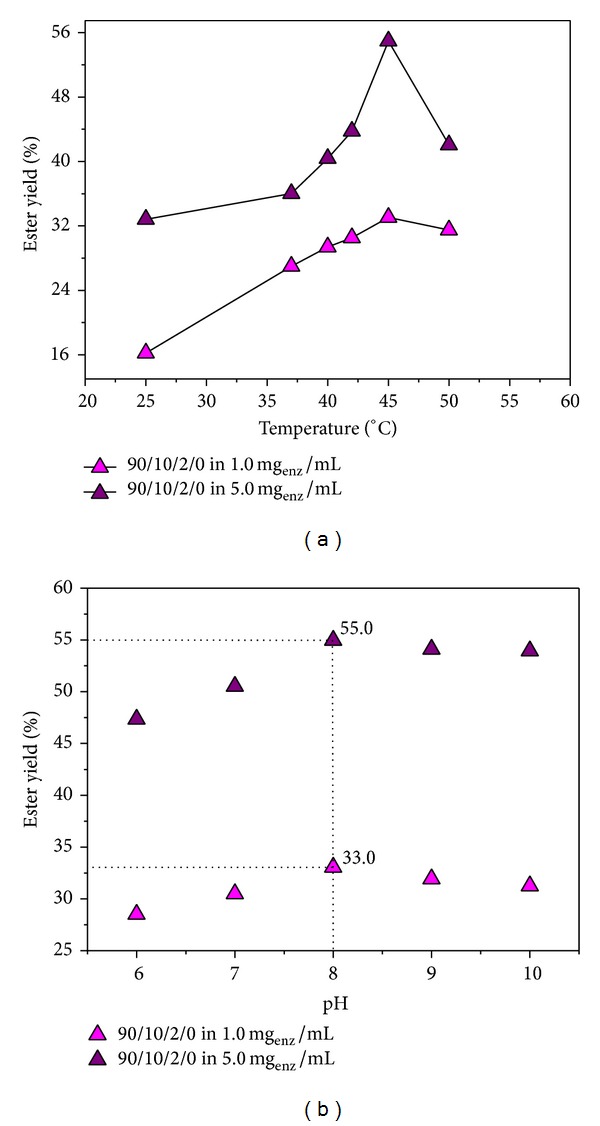
The effect of (a) temperature (200 *μ*L of pH 8.00 buffer solution, 0.2000 g of biocatalysts; cycle of 24 h) and (b) pH (45°C, cycle of 24 h; 0.2000 g of biocatalysts, 200 *μ*L of different buffer solutions) to the esterification reaction of isobutyric acid *n*-amyl alcohol in *n*-hexane.

As known, the lipase activity and thus the ester yield can be affected by changing pH of the medium in which the reaction is performed. The affect of pH on ester synthesis was investigated in the pH range from 6.0 to 10.0 (Figure 3(b)) at 45°C. Previous studies of the lipase immobilized on different supports have shown that at 45°C, lipase shows satisfactory activity and gives the reasonable ester yields [31–34]. As seen from figure, by changing the pH of the surrounding medium, the ester yields were not significantly affected. The yield increases with increasing pH from 6.0 to 8.0 and then decreases slightly. The highest ester yields were 33.0% and 55.0% at pH 8.0, when applying 1.0 and 5.0 mg_enz_/mL, respectively, and then slowly decrease.

Previous studies have shown that changing the reaction temperature can affect the lipase activity and thus the yield of ester [35]. Therefore, the effect of temperature on the ester yield has been investigated, as well. The temperature was varied in the interval from 25 to 50°C, and the experiments were performed at pH 8.0. The composition of the reaction system was as follows: biocatalyst/pH of 8.00/*n*-amyl alcohol/isobutyric acid/*n*-hexane. The mass of biocatalysts was kept constant and was 0.20 g.

Figure 3(a) shows the effect of temperature on the esterification reaction. The yield of ester at pH 8.00 was above 33.0% and 55.0% for the samples swollen in 1.0 and 5.0 mg_enz_/mL solution, showing good stability of the entrapped lipase. By increasing the temperature above 45°C, ester yield decreases probably due to thermal deactivation of enzymes. The good thermal stability of the enzyme after the entrapment is certainly expanding its potential use as biocatalysts. Therefore, the operating temperature should not exceed 45°C. The optimum pH was around pH 8.00.

**Figure 4:**
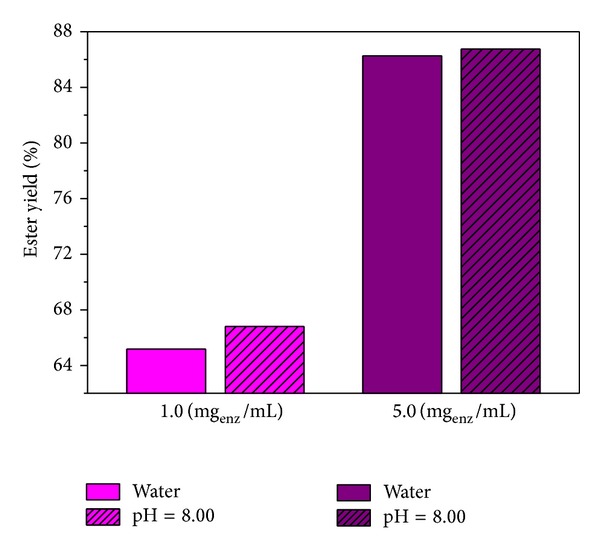
The effect of the initial lipase concentration in swelling solution on the ester yield in the esterification reaction of isobutyric acid and *n*-amyl alcohol in *n*-hexane, catalyzed by CRL (45°C, cycle of 24 h, 200 *μ*L of distilled water and buffer solution pH 8.00).

Figure 4 shows the effect of initial lipase concentration in swelling solution on the ester yield and it is clear that higher initial lipase concentration gives higher ester yields, as expected.

Finally, it can be concluded that the optimum temperature for esterification reaction was 45°C. The optimal pH for the reaction was around pH 8.00.

### 4.5. Swelling of Hydrogels in *n*-Hexane at 45°C

**Figure 5:**
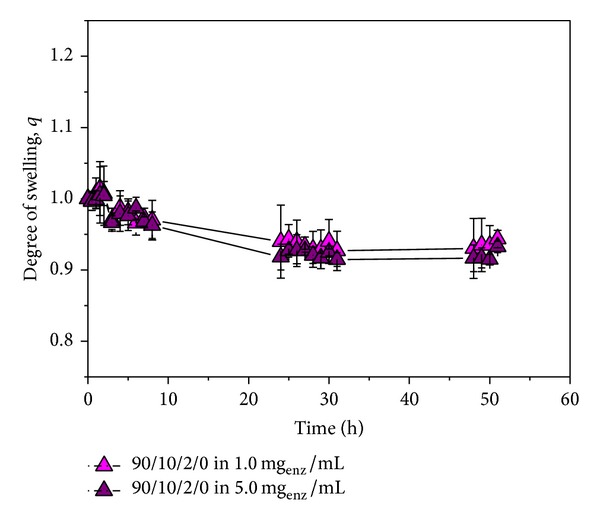
Swelling of hydrogels in *n*-hexane at 45°C.

A temperature of 45°C for sample swelling was selected since it was previously determined that this is the optimal temperature for ester synthesis. Since the esterification reaction was performed in *n*-hexane, it was necessary to examine the swelling of selected samples in this solvent at 45°C (Figure 5). As expected, the swelling of P(NiPAAm/IA) hydrogels was negligible in the hydrophobic solvent such as *n*-hexane at a temperature above the LCST values for PNiPAAm hydrogel.

The experiments of swelling in pure *n*-hexane indicated that the low water content is required for the esterification reaction. Water “triggers” the esterification reactions, known to take place at interfaces.

### 4.6. The Effect of Water Content on the Synthesis of *n*-Amyl Isobutyrate Catalyzed by the Entrapped CRL

As is known, water plays a key role in the lipase-catalyzed esterification reactions. Water participates in all noncovalent interactions that maintain open conformation of the catalytic center of lipase, and, on the other hand, the water content affects the conversion of the reaction [36]. When the esterification reaction takes place it is well known that with the increase of the water content lower conversion is achieved [37]. It is therefore necessary to determine the optimum water content but also the water content which allows excellent yields. The added water content is influenced by factors such as type of support, type of organic solvent and its content and biocatalysts content. The literature describes data for similar reactions in which the water content was in the range from 0.2 to 3.0 vol.% based on the dry enzyme [38].

**Figure 6:**
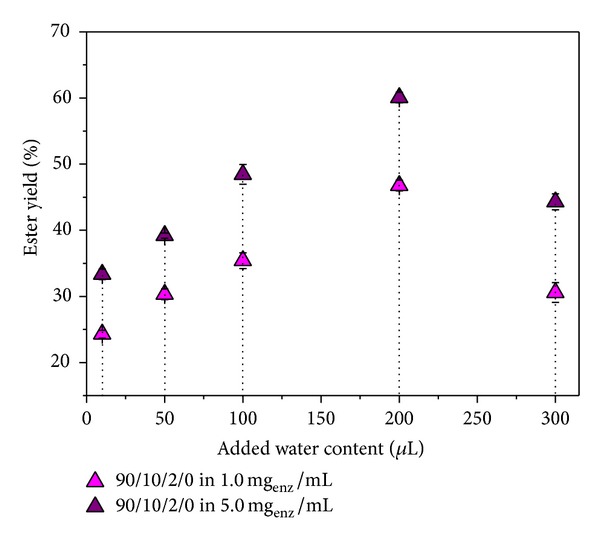
The effect of added water content on the esterification reaction in 1.0 and 5.0 mg_enz_/mL of CRL solution at 45°C (cycle of 48 h, 0.2000 g biocatalysts).

In this paper, the esterification reaction was performed, using different added water content in the range from 0.1 to 3.0 vol.%. According to the results (Figure 6), it was observed that the addition of water in the reaction mixture activated esterification reaction and that there is an optimum water content that gives the highest ester yield. The highest ester yield is obtained when the added water content was 2.0 vol.%.

Esterification reaction was performed at 45°C because it is the optimum temperature for ester yield. On the other hand, the hydrogel swelling capacity at this temperature is small. When having added water content greater than 2.0 vol.%, ester yields drop because water is directly involved in the reaction and inhibits the acid or alcohol [39].

### 4.7. Effect of the Initial Lipase Concentration in the Ester Synthesis

**Figure 7:**
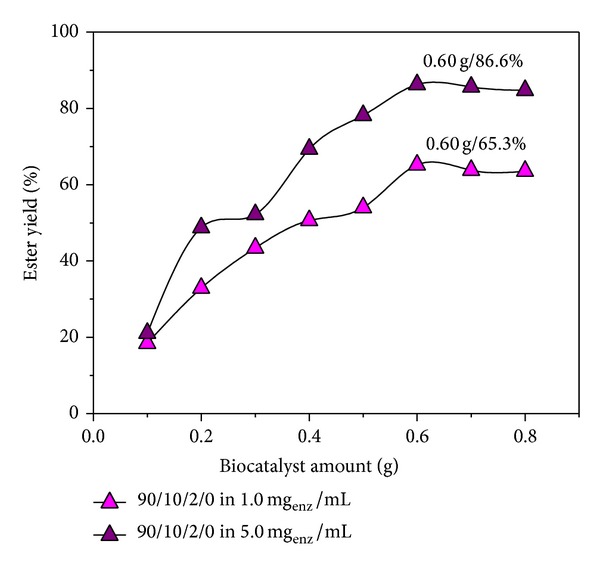
The effect of biocatalysts amount on the esterification reaction in solutions of different concentrations of lipase (a) 1.0 and (b) 5.0 mg_enz_/mL solution.

The effect of the mass of entrapped lipase on esterification reaction was investigated for a variety of biocatalysts content, from 0.10 to 0.80 g (equivalent to the enzyme mass of 0.0353 to 0.2822 g when the swelling was performed in 1.0 mg_enz_/mL of pH 7.0 and at 5°C and equivalent to the enzyme mass of 0.0230 to 0.1842 g, when the swelling was performed in 5.0 mg_enz_/mL of pH 7.0 and at 5°C). Added water content was constant (200 *μ*L). Obviously, the ester yields increase with increasing the biocatalysts amounts from 0.1 to 0.6 g and then slightly drop.  Figure 7 shows that the highest yield was obtained when the biocatalysts amount before the reaction was 0.6 g.

**Figure 8:**
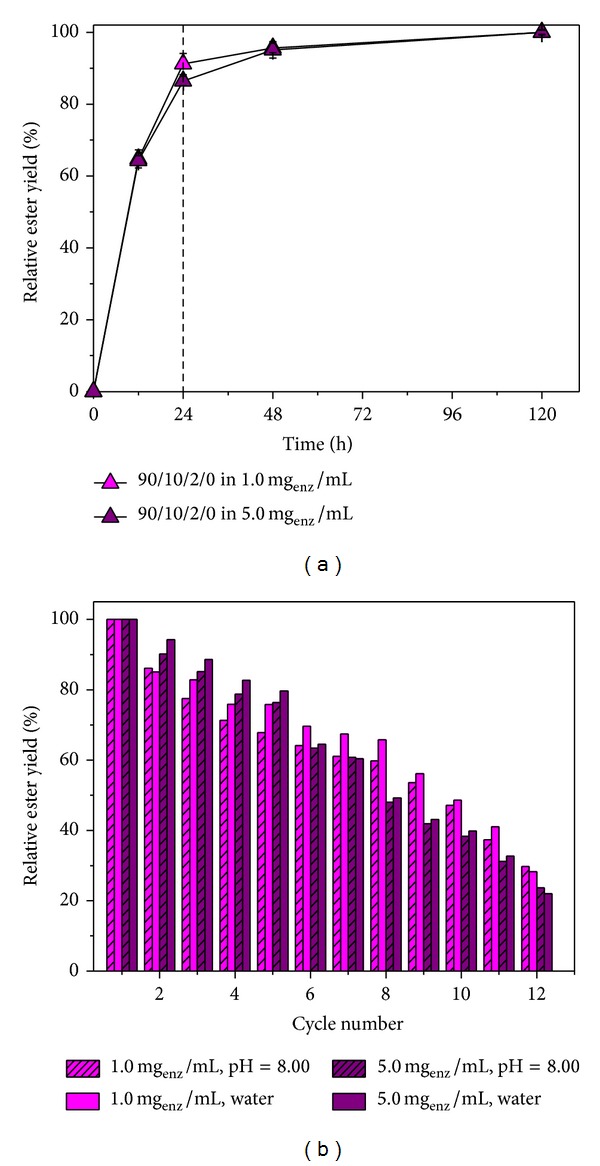
*n*-amyl isobutyrate formation by *Candida rugosa* lipase immobilized onto P(NiPAAm/IA) hydrogel (a) as function of time and (b) in repeated cycles at 45°C stirred at 150 rpm. Concentrations of isobutyric acid and *n*-amyl alcohol were 0.25 M; reactions were performed in *n*-hexane at fixed substrate molar ratio of 1.0.

The optimum reaction time was 24 h, after which the ester yield is practically constant (Figure 8(a)). It was shown that the esterification reaction rate and the conversion degree increased with increasing entrapped lipase amount.

Figure 8(a) shows the ester yield in the esterification reaction as a function of time, catalyzed by entrapped CRL at different lipase concentrations. Reaction half-time for hydrogels swollen in 1.0 mg_enz_/mL solution was about 9.5 h and when swollen in 5.0 mg_enz_/mL solution was about 9 hours, while complete consumption of biocatalysts occurs after almost 72 h at both concentrations.

### 4.8. Operational Stability and Repeated Use of Biocatalysts

The possibility of enzyme regeneration as a catalyst and its reuse is extremely important for the application of immobilized enzymes in industry [40] because recovery of immobilized enzymes lowers the price of final products and makes enzymatic processes economically viable. The esterification reaction catalyzed by lipase entrapped onto P(NiPAAm/IA) hydrogel was performed in 12 cycles. Each cycle lasted for 24 h, and between each cycle, the samples were dried in oven to constant weight. As shown in Figure 8(b), after first cycle, there is a significant loss of the ester yield. Esterolytic activity of the sample was maintained until 12th cycle. After 9th cycle, the ester yield decreased to 50%, also due to the loss of biocatalyst caused by the constant washing of the sample and/or impossibility to completely remove water during the drying of the sample.

The decrease in ester yields after a few cycles is probably due to loss of biocatalysts during filtration and drying process, while at the same time, higher amounts of byproduct/water were produced [41]. As presented, the applied lipase immobilization method more likely binds lipase to the hydrogel surface, making it more accessible to the substrate and reducing the esterification reaction time. However, this probably facilitates its separation from the surface by flashing, which reduces the reuse possibility of the catalyst. However, the operational stability of the entrapped lipase was good enough to be applied as biocatalyst in esterification reaction.

### 4.9. The Kinetic Study of *n*-Amyl Isobutyrate

In order to find adequate model for description of lipase-catalyzed *n*-amyl isobutyrate synthesis, initial reaction rates were determined for different concentrations of *n*-amyl alcohol and isobutyric acid. Ranges in which substrates were varied were chosen in accordance with literature survey and preliminary results [27].

As mentioned before, model which gave the best fit with experimentally obtained data was ping-pong bi-bi, when achieved goodness of fit was *R* ^2^ = 0.91. Proposed model was previously used for description of lipase-catalyzed synthesis of different aliphatic esters [27, 42], as well as in the recently reported ascorbyl oleate synthesis [43].

**Figure 9:**
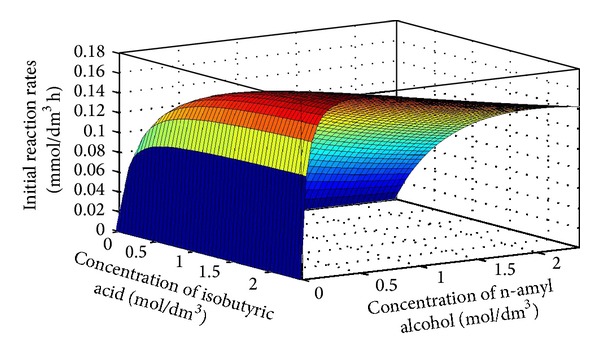
Appearance of the obtained ping-pong bi bi model with alcohol inhibition when xerogel was immersed in a CRL solution of concentration of 1 mg/dm^3^.

From the appearance of the 3D diagram (Figure 9), it could be observed that initial reaction rates increase with the increase of *n*-amyl alcohol up to concentration of 0.4 mol/dm^3^, while further increase of* n*-amyl alcohol led to decrease in initial reaction rates, as a repercussion of the biocatalyst inhibition with* n*-amyl alcohol. This effect was not observed with acyl donor, isobutyric acid, where increase in acid concentration led to continuous increase of initial reaction rates. Values of the obtained kinetic constants are presented in Table 1.

Comparison of the kinetic constants for *n*-amyl isobutyrate synthesis catalyzed with free and entrapped CRL [27] showed that entrapment of the enzyme led to small decrease in affinity towards acid (for free CRL *Km*_ac_ = 0.06 mol/dm^3^), while affinity towards alcohol increased.

From the comparison of the kinetic constants for synthesis *n*-amyl isobutyrate with entrapped and free CRL, it could be observed that entrapment of the CRL caused slight decrease in affinity towards isobutyric acid (for free CRL *Km*_ac_ = 0.06 mol/dm^3^). On the other hand, value of the Michaelis constant for alcohol (for free CRL *Km*_al_ = 0.75 mol/dm^3^) indicated that affinity of the entrapped CRL towards* n*-amyl alcohol increased in great deal. Also entrapment of the enzyme led to reduction of inhibition with *n*-amyl compared to free enzyme, since the value of the Inhibition constant (Table 1) was higher than one previously reported for free enzyme (*Km*_ia_ = 0.02 mol/dm^3^) [27].

## 5. Conclusion

The paper dealt with *Candida rugosa* lipase entrapment onto P(NiPAAm/IA) hydrogel and evaluated for both hydrolysis of olive oil in aqueous medium and *n*-amyl isobutyrate synthesis in organic medium. Although satisfactory biocatalytic performance in the aqueous system was achieved, the immobilized lipase was shown to be more suitable for application in ester synthesis in low aqueous system based on *n*-hexane. The esterification reaction parameters such as temperature, biocatalyst amount, initial water content, and substrate concentration on ester yield were investigated. The presented immobilized method could be applied in a wider range of temperature and pH and possess the enhanced reusability, thermal stability, and storage stability in comparison to free enzyme, which demonstrates that a stable three-dimensional structure of enzyme was created after immobilization. The operational stability of the immobilized system in esterification reaction proved to be fairly good with 8 consecutive 24 hours of uses with a residual activity of about 50%, implying that the developed hydrogel and immobilized system could provide a promising solution for the flavor ester synthesis at the industrial scale. Synthesis of the *n*-amyl isobutyrate catalyzed with entrapped CRL was adequately approximated with ping-pong bi-bi kinetic model with* n*-amyl alcohol inhibition. Values of the obtained kinetic constants indicate that entrapment of the CRL changed the affinity of the enzyme towards both substrates and reduced inhibition of the enzyme with *n*-amyl alcohol.

## Supplementary Material

Supplementary Materials include three tables providing the information on the effect of temperature, pH and lipase concentration in the solution on lipase activity under various conditions during the optimization process.

## Figures and Tables

**Table 1:** Values of kinetic constants from ping-pong bi-bi model with alcohol inhibition.

Parameter	Value
*V*_max⁡_ (mmol/dm^3^h)	0.22
*Km*_ac_ (mol/dm^3^)	0.1
*Km*_al_ (mol/dm^3^)	0.05
*K*_ial_ (mol/dm^3^)	0.21
