# Supplementary material for: Catalyzed Ester Synthesis Using Candida rugosa Lipase Entrapped by Poly(N-isopropylacrylamide-co-itaconic Acid) Hydrogel
Source: ScientificWorldJournal. 2014 Feb 20;2014:142123. doi: 10.1155/2014/142123 (PMC3950957; doi:10.1155/2014/142123)
Supplement: Supplementary file 1 — Supplementary Materials include three tables providing the information on the effect of temperature, pH and lipase concentration in the solution on lipase activity under various conditions during the optimization process. [file 142123.f1.pdf]

Supplementary Table 1. The effect of temperature on lipase activity after entrapment (xerogels swelling in lipase pH 7.00 ± 0.01 buffer solution of concentration of 1.0 mg_{enz}/mL).

Sample	***t = 5 °C***			***t = 25 °C***			***t = 37 °C***		
	***IU/g**$_{milled\ xerogel}$*	***IU/mg**$_{enz}$*	***Y, %***	***IU/g**$_{milled\ xerogel}$*	***IU/mg**$_{enz}$*	***Y, %***	***IU/g**$_{milled\ xerogel}$*	***IU/mg**$_{enz}$*	***Y, %***
85/15/2/0	34.20	0.171	28.5	30.60	0.153	25.5	21.20	0.106	17.7
90/10/2/0	29.40	0.147	24.2	25.80	0.129	21.5	10.60	0.053	8.8
95/5/2/0	24.20	0.121	20.7	16.20	0.081	13.5	5.20	0.026	4.3
100/0/2/0	23.60	0.118	19.7	10.80	0.054	9.0	2.00	0.010	1.7
85/15/4/0	31.40	0.157	26.2	24.80	0.124	20.7	15.60	0.078	13.0
90/10/4/0	28.60	0.143	23.8	17.20	0.086	14.3	8.20	0.041	6.8
95/5/4/0	21.20	0.106	17.7	10.80	0.054	9.0	3.60	0.018	3.0
100/04/0	9.80	0.049	8.2	5.40	0.027	4.5	0.60	0.003	0.5

Supplementary Table 2. The effect of buffer solution pH on the activity of entrapped lipase (xerogels swelling was performed at 5 ºC in lipase pH 7.00 ± 0.01 buffer solution of concentration of 1.0 mg_{enz}/mL).

pH	***Sample***	***IU/g$_{milled\ xerogel}$***	***IU/mg$_{enz}$***	***Y, %***
6.04	90/10/2/0	13.60	0.068	11.3
	95/5/2/0	6.40	0.032	5.3
	95/5/4/0	3.40	0.017	2.8
7.00	90/10/2/0	29.00	0.145	24.2
	95/5/2/0	24.80	0.124	20.7
	95/5/4/0	21.40	0.107	17.8
8.00	90/10/2/0	22.00	0.110	18.3
	95/5/2/0	20.00	0.100	16.7
	95/5/4/0	8.60	0.043	7.2
8.99	90/10/2/0	/	/	< 1.0
	95/5/2/0	/	/	< 1.0
	95/5/4/0	/	/	< 1.0

Supplementary Table 3. The effect of lipase concentration in the solution on the entrapped lipase activity (xerogels swelling was performed at 5 ºC and in a CRL solution of pH 7.00 ± 0.01).

CRL solution concentration, mg/mL	***Sample***	***IU/g$_{milled\ xerogel}$***	***IU/mg$_{enz}$***	***Y, %***
0.2	90/10/2/0	12.20	0.061	10.2
	95/5/2/0	10.80	0.054	9.0
	95/5/4/0	5.80	0.029	4.8
1.0	90/10/2/0	29.00	0.145	24.2
	95/5/2/0	24.80	0.124	20.7
	95/5/4/0	21.40	0.107	17.8
5.0	90/10/2/0	63.20	0.316	52.7
	95/5/2/0	39.60	0.198	33.0
	95/5/4/0	27.80	0.139	23.2
10.0	90/10/2/0	65.00	0.325	54.2
	95/5/2/0	46.00	0.230	38.3
	95/5/4/0	39.20	0.196	32.7
20.0	90/10/2/0	62.80	0.314	52.3
	95/5/2/0	45.40	0.227	37.8
	95/5/4/0	39.00	0.195	32.5
